# RETRACTION: The Efficacy of Nursing Interventions in Preventing Surgical Site Infections in Patients Undergoing Surgery for Congenital Heart Disease

**DOI:** 10.1111/iwj.70587

**Published:** 2025-04-18

## Abstract

**RETRACTION:** HeP. and HaiY. , “The Efficacy of Nursing Interventions in Preventing Surgical Site Infections in Patients Undergoing Surgery for Congenital Heart Disease,” International Wound Journal 21, no. 4 (2024): e14850, 10.1111/iwj.14850.38522429 PMC10961171

The above article, published online on 24 March 2024, in Wiley Online Library (http://onlinelibrary.wiley.com/), has been retracted by agreement between the journal Editor in Chief, Professor Keith Harding; and John Wiley & Sons Ltd. Following an investigation by the publisher, all parties have concluded that this article was accepted solely on the basis of a compromised peer review process. In addition, further investigation by the publisher found that the methods section lacks sufficient detail and is not supported by the references. Thus, the experimental procedure as described is not reproducible and not supported by previous work. The editors have therefore decided to retract the article. The authors did not respond to our notice regarding the retraction.

**RETRACTION:** P. He and Y. Hai , “The Efficacy of Nursing Interventions in Preventing Surgical Site Infections in Patients Undergoing Surgery for Congenital Heart Disease,” International Wound Journal 21, no. 4 (2024): e14850, 10.1111/iwj.14850.38522429 PMC10961171

The above article, published online on 24 March 2024, in Wiley Online Library (http://onlinelibrary.wiley.com/), has been retracted by agreement between the journal Editor in Chief, Professor Keith Harding; and John Wiley & Sons Ltd. Following an investigation by the publisher, all parties have concluded that this article was accepted solely on the basis of a compromised peer review process. In addition, further investigation by the publisher found that the methods section lacks sufficient detail and is not supported by the references. Thus, the experimental procedure as described is not reproducible and not supported by previous work. The editors have therefore decided to retract the article. The authors did not respond to our notice regarding the retraction.

